# A new approach for cytokinin isolation from Arabidopsis tissues using miniaturized purification: pipette tip solid-phase extraction

**DOI:** 10.1186/1746-4811-8-17

**Published:** 2012-05-17

**Authors:** Jana Svačinová, Ondřej Novák, Lenka Plačková, René Lenobel, Josef Holík, Miroslav Strnad, Karel Doležal

**Affiliations:** 1 Laboratory of Growth Regulators, Faculty of Science, Palacký University & Institute of Experimental Botany AS CR, v.v.i., Šlechtitelů 11, Olomouc, CZ-783 71, Czech Republic; 2 Department of Forest Genetics and Plant Physiology, Umeå Plant Science Centre, Swedish University of Agricultural Sciences, Umeå, SE-901 83, Sweden; 3 Centre of the Region Haná for Biotechnological and Agricultural Research, Faculty of Science, Palacký University, Šlechtitelů 11, Olomouc, CZ 783 71, Czech Republic; 4 Isotope Laboratory, Institute of Experimental Botany ASCR, v.v.i., Vídeňská 1083, Prague, 142 20, Czech Republic

**Keywords:** Cytokinins, *Arabidopsis thaliana*, Pipette tip solid-phase extraction (PT-SPE), StageTip, Ultra-high performance liquid chromatography (UHPLC), Tandem mass spectrometry (MS/MS)

## Abstract

**Background:**

We have developed a new analytical approach for isolation and quantification of cytokinins (CK) in minute amounts of fresh plant material, which combines a simple one-step purification with ultra-high performance liquid chromatography–fast scanning tandem mass spectrometry.

**Results:**

Plant tissue samples (1–5 mg FW) were purified by stop-and-go-microextraction (StageTip purification), which previously has only been applied for clean-up and pre-concentration of peptides. We found that a combination of two reverse phases and one cation-exchange phase, was the best tool, giving a total extraction recovery higher than 80%. The process was completed by a single chromatographic analysis of a wide range of naturally occurring cytokinins (bases, ribosides, *O*- and *N*-glucosides, and nucleotides) in 24.5 minutes using an analytical column packed with sub-2-microne particles. In multiple reaction monitoring mode, the detection limits ranged from 0.05 to 5 fmol and the linear ranges for most cytokinins were at least five orders of magnitude. The StageTip purification was validated and optimized using samples of *Arabidopsis thaliana* seedlings, roots and shoots where eighteen cytokinins were successfully determined.

**Conclusions:**

The combination of microextraction with one-step high-throughput purification provides fast, effective and cheap sample preparation prior to qualitative and quantitative measurements. Our procedure can be used after modification also for other phytohormones, depending on selectivity, affinity and capacity of the selected sorbents.

## Background

**Figure 1:**
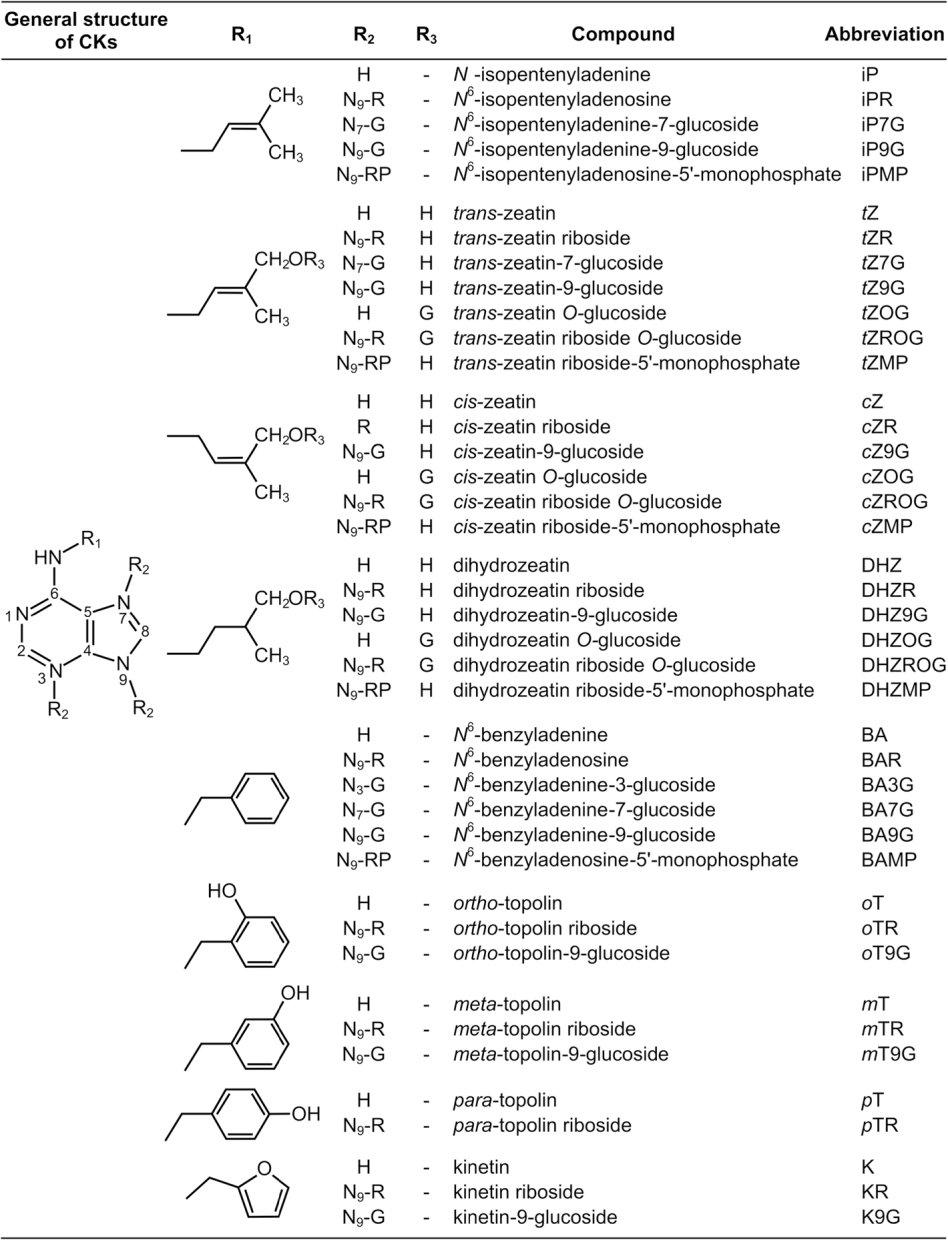
**Structures, names and abbreviations of the studied cytokinins.** H = hydrogen, R = *β-*D-ribofuranosyl, G = *β-*D-glucopyranosyl, RP = *β-*D-ribofuranosyl-5^′^-monophosphate

The plant hormone cytokinin (CK) is essential to promote cell growth and differentiation in plant tissues in the presence of another phytohormone, auxin [1-3]. Natural cytokinins are adenine derivatives, which are substituted at the *N*^*6*^ position by either an isoprenoid or an aromatic side chain (Figure 1). The presence of a hydroxyl group, double bonds and other structural variations affect cytokinin-receptor interactions and determine the functional specificity of individual CKs [4,5]. Cytokinins occur in plants as free bases, nucleosides (ribosides), glycosides (*O*- and *N*-glycosides) and nucleotides. The occurrence, distribution and variation of individual CKs depend on plant species, tissue, and developmental stage [2,3]. 

The analysis of cytokinins is difficult because of their presence in plants at very low concentrations (pmol g^−1^ fresh weight). Modern analytical procedures for the determination of cytokinins consist of sample pre-treatment and subsequent instrumental measurement of individual CK metabolites [6]. Extraction of analytes from the plant material by an organic solvent such as Bieleski buffer (methanol/chloroform/water/formic acid, 12/5/2/1) [7], 80% (v/v) methanol or modified Bieleski buffer (methanol/water/formic acid, 15/4/1) [8] is usually followed by solid-phase extraction (SPE) using reversed-phase, cation- and anion-exchange sorbents [9-11]. The final step for isolation of biologically active compounds from plant extracts often involves an immunoaffinity chromatography [12,13]. During the last decade, the starting amount of plant material has decreased from gram (1–5 g) to milligram quantities (50–100 mg) [13-15]. The rapid technical development of mass spectrometers has provided technology to detect plant hormones in *mg* amounts of plant tissues [16-18]. For determination of auxin in small samples (0.05–0.1 mg of tissue), a technical level was reached where losses of reproducibility due to factors such as contamination and precision in tissue sampling can pose equal or even larger problems than the analytical sensitivity *per se*. This is still not the case for several other phytohormones, for which improvements in instrumentation and sample purification would greatly facilitate attempts to address questions being asked in developmental biology [18]. To minimize the required amount of biological tissues (<5 mg FW), the pipette tip SPE (PT-SPE) method is a useful tool for purification, concentration, and selective compound isolation. The PT-SPE method, in which chromatographic material is polymerized into pipette tips, is mainly used in genomic, proteomic and biomedical applications [19-22]. However, no previous applications of PT-SPE for phytohormone analysis have been reported. The so-called StageTip (STop And Go Extraction Tip) purification has been published as a simple and extremely economical approach to desalting, concentration, purification and pre-fractionation of proteins and peptides prior to LC/MS analysis [23-25]. StageTips are ordinary pipette tips containing very small disks made of beads with reversed-phase or ion-exchange surfaces embedded in a Teflon mesh [26]. These self-made micro-tips combine flexibility, ease and speed of use, small bed volume and high loading capacity, and provide excellent recovery, high reproducibility, robustness and versatility for even minute amounts of analyte. A further advantage for sample preparation is that the one-time usage eliminates the risk of carryover [26].

High performance liquid chromatography (HPLC) combined with mass spectrometric (MS) detection has become the main technique of choice in cytokinin analysis after pre-cleaning samples [10,14,27]. The introduction of ultra-high performance liquid chromatography (UHPLC), in which sub-2-microne particles are packed in a column, brought new improvements compared with HPLC in terms of separation efficiency, resolution, sensitivity, speed and sample throughput [28-30]. Fast chromatographic separation of complex samples and low concentration of analytes require a sensitive fast scanning tandem mass spectrometer (MS/MS) which provides a trace analysis of femtomolar concentrations. The practical application of UHPLC-MS/MS methods has been reported for different phytohormone groups [13,16,31-34]. Recently, miniature sample pretreatments based on hydrophilic interaction chromatography combined with MS/MS were used for analysis of cytokinins [17,35].

The main aim of this study was miniaturization of cytokinin isolation method with applicability to plant tissue samples weighing as little as 1–5 mg fresh weight. To achieve this, we have successfully optimized the purification step employing self-packed StageTips as a novel powerful PT-SPE approach to complex cytokinin analysis. Finally, we have developed new UHPLC-MS/MS methods requiring only a single chromatographic run. The improved method uses an efficient separation onto a C18 column packed with sub-2-microne particles, combined with accurate quantification by MS/MS in multiple reaction monitoring modes (MRM) for measurement of naturally occurring isoprenoid cytokinin metabolites (bases, ribosides, *N*-glucosides, *O*-glucosides and nucleotides).

## Results and discussion

### Development of StageTip purification method

**Figure 2:**
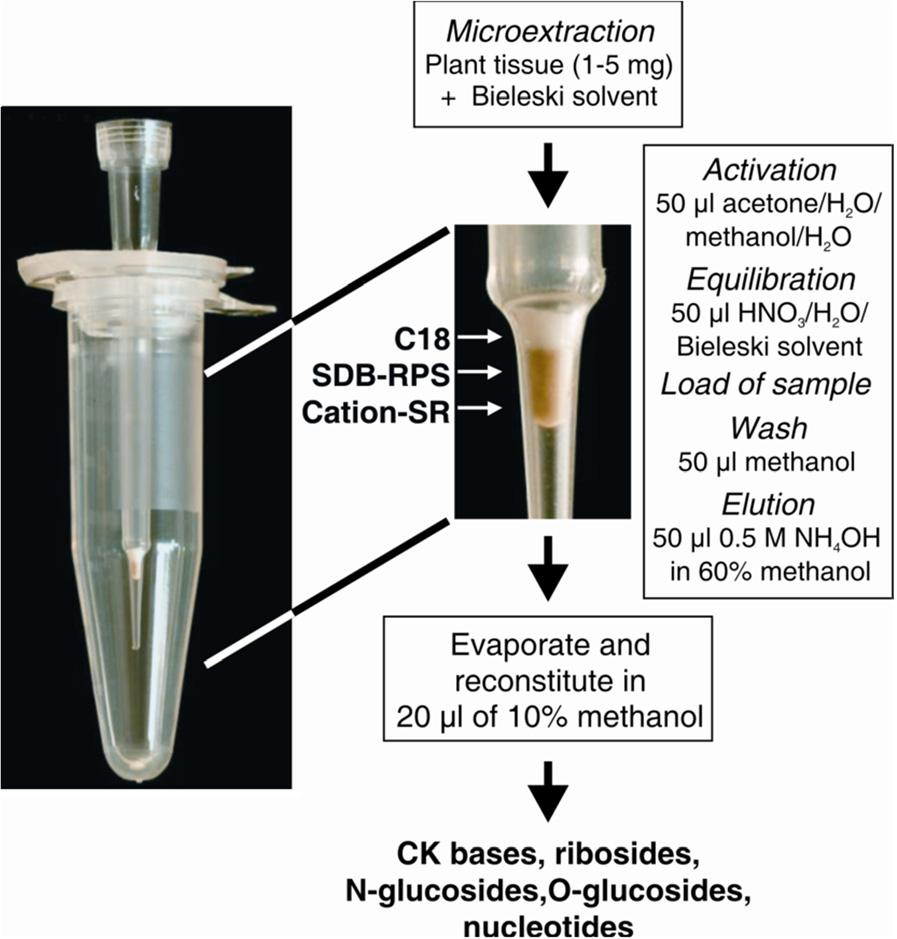
**Purification protocol for cytokinins using multi-StageTips (STop And Go Extraction Tips).** Three different sorbents (C18/SDB-RPS/Cation-SR) were placed in an ordinary pipette tip and inserted into a microcentrifuge tube (1.5 ml). Plant material (1–5 mg) was homogenized and extracted in Bieleski buffer containing labelled internal standards. Pooled supernatant was applied (1 mg FW 50 μl^–l^) to a pre-conditioned multi-StageTip microcolumn, which was then washed and eluted with the indicated solutions. The eluate was evaporated to dryness, dissolved in 20 μl of 10% methanol and analyzed by UHPLC-MS/MS method.

The optimization of homogenization, extraction and purification, critical and rate-limiting steps in the isolation of natural compounds, is particularly important for small sample sizes, where losses and cross-contamination must be reduced to a minimum. In SPE, results depend strongly on the physical-chemical properties of the analytes, as well as on the kind of sample matrix and the choice of sorbent which can control parameters such as selectivity, affinity and capacity [30]. The characteristics of different sorbents can be determined by measuring the recovery of labelled standards, a mixture of which is added to the sample during the purification procedure. For this purpose, we chose the tritium-labelled cytokinins (^3^H]*c*Z, ^3^H]*t*ZR, or ^3^H]iPR) representing compounds with different polarities and retention times under our chromatographic conditions (see below). The charge on cytokinin molecules may be positive or negative depending on the pH (changes from pH < 3 to pH > 11 result in the charge becoming progressively more negative). Moreover, the presence of an *N*^*6*^-side chain adds hydrophobicity to molecules such as adenine [9]. In the highly acidic extraction mixture, cytokinins should be predominantly presented as cations, and they should be completely retained on a cation-exchange sorbent from which they can be eluted with an alkaline solvent. The presence of an organic constituent in Bieleski buffer (60% MeOH) reduces the ability of non-polar compounds to be retained on a reversed-phase sorbent. These theoretical expectations were confirmed by a test of micropurification protocol (Figure 2) using single-StageTips filled with different sorbents (C18, SDB-RPS, and Cation-SR). The recoveries of added ^3^H-CK standards (10^5^ dpm) in Bieleski buffer applied onto microcolumns are summarized in Table 1. In agreement with our previous findings, the most hydrophobic cytokinin tested, iPR, was not retained at all on C18 sorbent following the direct application of 50 μl acidic extract. This illustrates that higher concentrations of organic solvent (in combination with acidic conditions) may influence the selectivity of octadecyl-bonded silica Empore Disks. A high loading capacity was observed while using the second reversed-phase sorbent (SDB-RPS) based on poly(styrene-divinylbenzene) copolymer modified with sulfonic acid groups, which retains analytes by different mechanisms such as hydrophilic, hydrophobic and π-π interactions [36]. Moreover, the extraction recoveries were comparable to the cation-exchange sorbent (Table 1). 

**Table 1:** The loading capacity and extraction recovery of single-StageTip microcolumns

	**Recovery of**^**3**^ **H-labelled CKs (%)**					
	**C18**		**SDB-RPS**		**Cation-SR**	
	**Flow through**	**Elution**	**Flow through**	**Elution**	**Flow through**	**Elution**
[^3^H]*c*Z	98.8 ± 3.3	0.6 ± 0.2	1.3 ± 0.1	96.6 ± 1.8	1.1 ± 0.1	100.7 ± 3.0
[^3^H]*t*ZR	98.8 ± 4.4	0.6 ± 0.2	39.2 ± 2.2	29.8 ± 2.2	22.4 ± 0.6	64.7 ± 1.1
[^3^H]iPR	102.9 ± 3.5	0.5 ± 0.3	4.9 ± 0.2	95.6 ± 1.4	4.7 ± 0.1	97.0 ± 2.8

Values are means ± SD (n = 4).

Recovery (%) of ^3^ H-labelled CK standards without added plant matrix. Tritium-labelled standards (10^5^ dpm) were dissolved in Bieleski buffer and applied onto different Empore sorbents (C18, SDB-RPS, Cation-SR).

**Figure 3:**
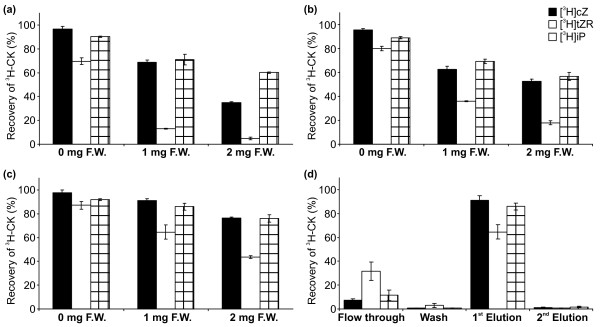
**The loading capacity and extraction recovery of multi-StageTip microcolumns.** (**a-c**) Recovery (%) of ^3^H-labelled CK standards in the first elution step (50 μl 0.5 M NH_4_OH in 60% MeOH) applied onto multi-StageTips using Empore sorbents in different combinations, C18/SDB-RPS (**a**), C18/Cation-SR (**b**), C18/SDB-RPS/Cation-SR (**c**) without (0 mg FW) and with plant matrices (1 or 2 mg FW). (**d**) Representative test of loading capacity and extraction recoveries at different steps during the purification protocol described in Figure 2 for 1 mg FW of *A. thaliana* seedlings extracted. For all experiments, tritium-labelled standards (10^5^ dpm) were dissolved and the extracts were prepared in Bieleski buffer. Values are means ± SD (n = 4); [^3^H]*c*Z, black bars; [^3^H]*t*ZR, white bars; [^3^H]iPR, square bars.

Due to our presumption of the strong matrix effect and the possibility of easy preparation of various multi-StageTips, we also tested the combination of reversed-phase and/or cation-exchange sorbents, C18/SDB-RPS, C18/Cation-SR, C18/SDB-RPS/Cation-SR, respectively. The PT-SPE purification procedure was optimized using two types of sample in quadruplicate: (i) samples containing one of the selected ^3^H-labelled standards (10^5^ dpm) in extraction buffer; and (ii) *A. thaliana* seedling extracts prepared from 1.0 and 2.0 mg FW with added ^3^H-labelled cytokinin standards. For both sample types, the recoveries of ^3^H-CKs were determined (Figure 3). By utilizing different types of stationary phase, cations as well as hydrophobic compounds should be retained in accordance with results published by Dobrev et al. [9] who developed a method for separation and purification of cytokinins from auxin and abscisic acid using commercially available mixed-mode SPE columns. As demonstrated in Figure 3, more cytokinin standards were recovered from the samples without added plant extract. The presence of plant matrix in the sample caused slightly decreased yields of the added CK standards; however the values were still close to 80%. The effect of plant matrix was also obvious from the correlation between the increasing sample weight and decreasing recovery values. Under our experimental conditions (Figure 2), all tested tritium-labelled standards were eluted in the first elution step by 50 μl of 0.5 M NH_4_OH in 60% MeOH. For instance, the combination of C18/SDB-RPS sorbents used for the purification of spiked 1 and 2 mg plant extracts recovered 68 ± 2% and 36 ± 1% for ^3^H]*c*Z, 13 ± 1% and 5 ± 1% for ^3^H]*t*ZR, 78 ± 4% and 70 ± 2% for ^3^H]iPR, respectively (Figure 3a). Despite our reasoning and previous experience [10,13], the replacement of a polymer based sorbent (SDB-RPS) by cation-exchange phase (Cation-SR) in combination with plant matrices did not greatly improve the selectivity and retention of cytokinins on multi-StageTips (Figure 3b). Hence, the combination of all three sorbent types (C18/SDB-RPS/Cation-SR) in one multi-StageTip was examined and final recoveries for each of the tested ^3^H-CK showed the highest efficiency in the purification procedure (Figure 3c). Moreover, radioactivity of tritium-labelled CKs was measured continuously at different steps in the tested purification protocols (flow through, wash and elution) to monitor the loading capacity and extraction recovery (Figure 3d). For the definite StageTip configuration, the high selectivity and acceptable retention of all analytes were demonstrated by minute losses in the methanolic wash and second elution step, respectively. However, the losses of all ^3^H-CK standards in loading step depend on increasing sample amount and are in the range 7–31% and 21–53% of tritium-labelled CKs for 1.0 and 2.0 mg FW, respectively (see Figure 3d; Additional file 1). The results confirmed that the triple combination of sorbents in StageTips was the best tool in the new miniaturized one-step purification procedure. In conclusion, a total extraction recovery higher than 80% and reproducibility with an RSD of 10% (or less) was in good agreement with previously published data using PT-SPE for analysis of drugs in different biological matrices [21,22]. 

### Development of the UHPLC-ESI(+)-MS/MS method

**Figure 4:**
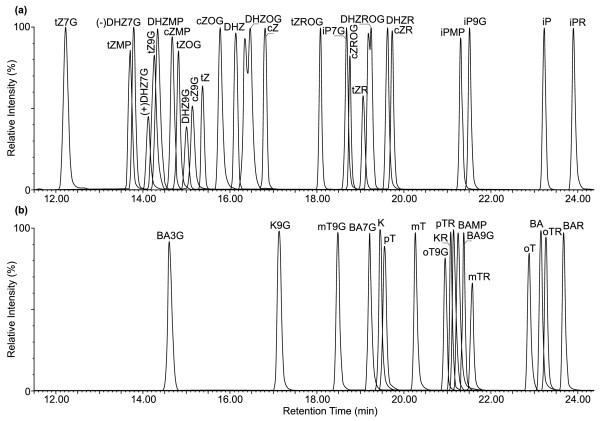
**Separation of cytokinin standards by UHPLC-ESI(+)-MS/MS method using an Acquity UPLC® BEH C18 2.1 × 150 mm column.** Multi-MRM chromatograms of 25 isoprenoid cytokinins (**a**) and 17 aromatic cytokinins (**b**) including bases, ribosides, *N-/O-*glucosides and nucleotides containing 1 pmol of each derivative per injection.

Development of the new procedure, which includes only one-step high-throughput solid-phase extraction, also required the selection of appropriate analytical conditions, namely the higher separation efficiency and resolution of a UHPLC system together with maximal selectivity and sensitivity of a tandem MS instrument. The complex plant matrix, and presence of unidentified peaks in the individual mass chromatograms complicate the final selectivity of tandem MS detection [14]. An additional issue which can further complicate the separation is that protonated CK metabolites ([M + H]^+^) produce a typical fragmentation pattern after collision induced dissociation, characterized by the loss of the nucleosyl/glycosyl/nucleotyl group and the fragmentation of the *N*^*6*^-substituent for the aglycons (Table 2). Moreover, since an in-source fragmentation of ribosides occurs, the signal of the aglycone is obscured by the signal of its riboside in cases where they co-elute [37]. In agreement with this observation, analogous in-source dissociations were observed for all CK derivatives. Therefore a separation with higher peak resolution was preferred to obtain accurate endogenous levels of free bases, nucleosides (ribosides), glycosides (*N*- and *O*-glucosides) and nucleotides. The chromatographic conditions using methanol/15 mM HCOOH (pH 4.0, adjusted by NH_4_OH) for a fast cytokinin analysis [13] were modified here to obtain the baseline separation of 25 isoprenoid *N*^*6*^-adenine isomers onto reversed-phase column packed with sub-2-microne particles (Figure 4). Moreover, a change in column length can also increase the resolution of the critical pair of isomer peaks. We have tested three different BEH C18 column lengths, viz. 50 mm, 100 mm, and 150 mm. The shorter columns were unable to resolve all the compounds of interest, especially the closely eluted pairs such as *c*Z9G/*t*ZOG, *c*Z/*t*ZROG, and DHZ/DHZOG (data not shown). With the optimized isocratic steps and programme of gradients, glycosylated zeatin-types (*t*Z7G, *t*Z9G, *c*Z9G, *t*ZOG,and *c*ZOG) were subsequently eluted with different retention times (12.26 ± 0.02, 14.23 ± 0.02, 15.13 ± 0.02, 14.83 ± 0.03 and 15.79 ± 0.03 min). Finally, we compared the high resolution of the LC method with retention of the aromatic CK group, which is characterized by its higher hydrophobicity (Figure 4b). Under the optimized conditions, all bases, ribosides and 9-glucosides of *ortho-meta-*, and *para*-topolins, as well as benzyladenine and kinetin with corresponding derivatives, were baseline separated. Although the final separation of seventeen aromatic cytokinins was not the main purpose of this work, the method we describe here could be adapted for their analysis. 

**Table 2:** Method parameters

**Compounds**	**Precursor**	**Products**	**Retention time**^**a**^**(min)**	**LOD**^**b**^**(fmol)**	**Dynamic range (mol)**	***R***^***2***^
t/cZ	220.1	**136.1**, 119.0	15.39 ± 0.04/16.82 ± 0.03	0.5	1×10^−15^–5×10^−11^	0.9989/0.9987
t/cZR	352.2	**220.1**, 136.1	19.06 ± 0.02/19.72 ± 0.01	0.1	5×10^−16^–5×10^−11^	0.9993/0.9986
tZ7G	382.2	**220.1**, 136.1	12.26 ± 0.02	0.1	5×10^−16^–1×10^−11^	0.9989
t/cZ9G			14.23 ± 0.02/15.13 ± 0.02	0.1	5×10^−16^–5×10^−11^	0.9993/0.9985
t/cZOG			14.83 ± 0.03/15.79 ± 0.03	0.5	1×10^−16^–1×10^−11^	0.9987/0.9988
t/cZROG	432.2	**382.2**, 220.1	18.08 ± 0.02/18.77 ± 0.02	1.0	5×10^−15^–1×10^−11^	0.9992/0.9984
t/cZMP	514.2	**220.1**, 136.1	13.72 ± 0.02/14.67 ± 0.02	5.0	1×10^−14^–5×10^−11^	0.9990/0.9985
DHZ	222.1	**136.1**, 119.0	16.15 ± 0.04	0.1	5×10^−16^–1×10^−11^	0.9991
DHZR	354.2	**222.1**, 136.1	19.61 ± 0.01	0.05	1×10^−16^–5×10^−10^	0.9989
DHZ7G	384.2	**222.1**, 136.1	13.80 ± 0.02/14.13 ± 0.02	0.1	5×10^−16^–1×10^−10^	0.9994
DHZ9G			15.00 ± 0.01	0.05	1×10^−16^–1×10^−10^	0.9992
DHZOG			16.37 ± 0.03	0.1	5×10^−16^–5×10^−12^	0.9992
DHZROG	434.2	**384.2**, 222.1	19.22 ± 0.03	1.0	5×10^−15^–1×10^−11^	0.9983
DHZMP	516.2	**222.1**, 136.1	14.34 ± 0.01	1.0	5×10^−15^–1×10^−11^	0.9992
iP	204.1	**136.1**, 119.0	23.21 ± 0.01	0.1	5×10^−16^–1×10^−11^	0.9991
iPR	336.2	**204.1**, 136.1	23.88 ± 0.01	0.05	1×10^−16^–1×10^−11^	0.9989
iP7G	366.2	**204.1**, 136.1	18.70 ± 0.01	0.05	1×10^−16^–1×10^−11^	0.9988
iP9G			21.50 ± 0.01	0.5	1×10^−15^–1×10^−11^	0.9992
iPMP	416.2	**204.1**, 136.1	21.31 ± 0.02	5.0	1×10^−14^–5×10^−11^	0.9993

^a^ Values are means ± SD (n = 10); ^b^ Limit of detection, defined as a signal-to-noise ratio of 3:1.

List of precursor and product ions of the studied compounds (the most abundant product ion is shown in bold type). The retention time stability, limits of detection, dynamic linear range, linearity (coefficient of determination, R^2^) are shown for UHPLC-ESI(+)-MS/MS analysis of isoprenoid cytokinins.

The higher efficiency of applied BEH technology using a long Acquity UPLC® reversed-phase column was also demonstrated by the separation of racemic (±)-dihydrozeatin derivatives. Moreover, the (−)-antipode was isolated as a natural cytokinin from immature seeds of *Lupinus luteus*[38]. The different cytokinin activity of enantiomers of unnatural (R)- and naturally-occurring (S)- configurations in dihydrozeatin and their ribosides was reported by Matsubara et al. [39]. Compared with a previously published LC method [40], N_7_-substituted glucosides, (±)-DHZ7G, were baseline separated (Figure 4a). Moreover, (±)-DHZOG and (±)-DHZROG were partially separated in 16.5 and 19.2 min, respectively, while (±)-DHZ, (±)-DHZR, and (±)-DHZ9G were still eluted as identical peaks. Possibly, changes in the orientation of the side chain resulting from *N-* or *O-*glycosyl substitution of the dihydrozeatins might interact differently with the stationary phase by the regular non-polar interactions with the aliphatic chains under the chromatographic conditions used. In Arabidopsis extracts, DHZ7G was identified as the peak with the highest intensity using the selective MRM transition (384.2 > 222.1) and comparing the retention time (13.78 ± 0.03 min) with injected standard (13.80 ± 0.02) under the same chromatographic conditions ( Additional file 2). In accordance with our results and the published natural occurrence of (S)-(−)-dihydrozeatins, we proposed that the first separated peak was (−)-DHZ7G with (S)-configuration. Moreover, we also confirmed the similar chromatographic behaviour for the other DHZ-*O*-glucoside derivatives.

The total run time of developed UHPLC-ESI(+)-MS/MS method, including equilibration, was 30 min and might be acceptable for a routine single chromatographic analysis of CK derivatives in biological samples. Under these compromise conditions, the retention times for the monitored compounds ranged from 12.0 to 24.5 min. Based on the excellent stability of the retention times, which ranged between 0.04% and 0.24% RSD (Table 2), the chromatographic run was split into five retention windows (11.0–13.0, 13.0–17.7, 17.7–20.0, 20.0–22.0, 22.0–24.5 min) which increased the sensitivity of the subsequent ESI-MS/MS measurements. Electrospray capillary and cone voltages were subsequently optimized to generate the required precursor ions in the positive ion mode and the collision energy was tuned to dissociate and produce characteristic fragments for each cytokinin. All precursor and product ions of unlabelled and labelled cytokinin types determined by UHPLC-ESI(+)-MS/MS as well as cone voltages (21–35 V) and collision energies (15–24 eV) corresponded with the previously published data [13].

### Validation of one-step purification and quantification method

In the first instance, for the improved UHPLC-ESI(+)-MS/MS method, the basic validation characteristics were calculated after repeatedly injecting solutions with varied concentrations of each unlabelled analyte and fixed concentrations of the corresponding IS. Limits of detection (LOD) and quantification (LOQ), based on 3:1 and 10:1 signal to noise ratios, ranged between 50 amol – 1 fmol and 100 amol – 5 fmol for all of the investigated analytes, respectively (Table 2). In accordance with our published fast UHPLC-MS/MS method, the lowest LODs were obtained for DHZR and iPR as well as for DHZ9G and iP7G, respectively. The highest LODs for cytokinin nucleotides correlated with the reduction in ionization efficiency associated with presence of phosphate groups on adenosine moiety [41]. The calibration curves constructed from 14 points were obtained from separate injections of standard mixtures and gave a broader linear range, spanning at least four orders of magnitude. For most cytokinins, the response was linear up to 10 pmol injected with the coefficients of determination (*R*^*2*^) varying from 0.9983 to 0.9994 (Table 2). The use of UHPLC-ESI(+)-MS/MS method with Scanwave technology [42] provided 5–25-fold greater sensitivity in MRM mode in comparison with previously published UHPLC-MS/MS techniques [13,33]. The linearity of the method was also in a good agreement with that of other authors using tandem mass spectrometry for cytokinin analysis [17,27,43,44].

For verification of the sample matrix effects, the efficiency of whole developed approach was evaluated by spiking 10-day-old Arabidopsis seedling extract with authentic and stable isotope-labelled cytokinin standards, 1.0 and 0.5 pmol added respectively (Table 3). The capacity of the StageTip microcolumns to isolate cytokinins from plant extracts was tested by purification of three different amounts of fresh plant weight (1.0, 2.0 and 5.0 mg) in quadruplicates. Recoveries were calculated as a percentage of defined amounts added to the sample prior to the StageTip purification procedure (Figure 2). After the PT-SPE step followed by chromatographic separation and mass spectrometric quantitative measurement, recoveries of cytokinins in the presence of plant matrices reached, on average, 77 ± 17%, 46 ± 17% and 10 ± 6% for 1.0, 2.0, and 5.0 mg FW, respectively (Table 3). These data are consistent with results obtained from the preliminary experiment with ^3^H-labelled CK standards (Figure 3). However, the other reason for decreased recovery in case of one-layer sorbent could be overload of the sorbents. We tested this hypothesis using multi-StageTip microcolumns packed with one-, two- and three-layers of each sorbent type (C18, SDB-RPS and Cation-SR). From the results in Table 4, it can be concluded that increasing the amount of sorbents effectively retains matrix interfering agents and improves the total recovery of developed PT-SPE protocol. The data also showed the increasing amount of the applied sample tissues as the main limitation for using of the multi-StageTips packed with one-layer sorbent as a high-throughput purification step. On the other hand, the yields for 1.0 mg indicated a 2-fold higher capacity of the SPE step than the previously published batch immunoextraction purification procedure, for which average values of 40% and 60% were found for isoprenoid and aromatic cytokinins, respectively [13]. Interestingly, behaviour of the cytokinins during the PT-SPE procedure was also highly dependent on their structural modification (conjugation) (Tables 4 and 5). This could be related to combination of two different mechanisms, the reversed-phase and cation-exchange, which are responsible for the retention of cytokinins on mixed-mode sorbents [9]. Independently on sample size, the highest recovery of CK-bases was obtained, followed by a lower yield of *N*-glucosides, *O*-glucosides, and ribosides, respectively. The lowest recovery was determined for CK-nucleotides which are relatively polar molecules with possibility to have one positive and one negative charge [41]. 

**Table 3:** Method validation

**CKs**	**Recovery (%)**^a^			**Determined spiked CK content (pmol)**^b^	**Method precision (% RSD)**^**b**^	**Method accuracy (% bias)**^**b**^
	**1 mg FW**	**2 mg FW**	**5 mg FW**			
*t*Z	80 ± 10	63 ± 8	21 ± 4	0.99 ± 0.17	17.2	0.1
*t*ZR	72 ± 12	46 ± 7	8 ± 1	0.84 ± 0.09	10.4	16.1
*t*Z7G	88 ± 6	57 ± 4	6 ± 1	0.91 ± 0.13	14.6	8.6
*t*Z9G	59 ± 7	31 ± 3	8 ± 1	0.97 ± 0.09	9.6	2.5
*t*ZOG	85 ± 5	68 ± 6	11 ± 2	1.07 ± 0.19	18.1	−7.1
*t*ZROG	55 ± 4	30 ± 3	4 ± 1	0.82 ± 0.09	11.1	17.6
*t*ZMP	35 ± 6	11 ± 1	5 ± 1	0.85 ± 0.11	13.3	14.7
*c*Z	75 ± 9	65 ± 5	24 ± 4	0.83 ± 0.02	2.5	17.0
*c*ZR	81 ± 13	44 ± 9	8 ± 1	0.96 ± 0.12	12.8	4.1
*c*Z9G	74 ± 12	37 ± 5	5 ± 1	1.18 ± 0.13	11.3	−17.6
*c*ZOG	89 ± 6	66 ± 7	9 ± 2	1.09 ± 0.14	13.2	−9.1
*c*ZROG	52 ± 6	24 ± 2	3 ± 1	0.89 ± 0.11	12.9	11.2
*c*ZMP	32 ± 3	17 ± 1	2 ± 1	0.86 ± 0.15	17.9	13.9
DHZ	77 ± 13	61 ± 7	20 ± 3	0.90 ± 0.10	10.8	9.7
DHZR	88 ± 13	48 ± 8	12 ± 1	1.03 ± 0.06	5.6	−2.9
DHZ7G	89 ± 3	65 ± 3	8 ± 2	1.18 ± 0.05	4.3	−18.2
DHZ9G	78 ± 10	35 ± 6	6 ± 1	0.96 ± 0.07	7.2	3.7
DHZOG	77 ± 5	50 ± 7	9 ± 3	1.16 ± 0.06	4.9	−15.8
DHZROG	87 ± 8	42 ± 5	5 ± 1	0.90 ± 0.12	13.3	9.8
DHZMP	37 ± 1	12 ± 1	3 ± 1	0.96 ± 0.15	15.8	3.6
iP	76 ± 9	68 ± 3	26 ± 5	0.97 ± 0.06	5.9	3.3
iPR	84 ± 8	53 ± 4	17 ± 1	1.13 ± 0.06	5.6	−12.8
iP7G	83 ± 10	60 ± 5	7 ± 1	0.91 ± 0.15	16.5	9.4
iP9G	74 ± 8	49 ± 8	8 ± 2	0.97 ± 0.08	8.0	3.1
iPMP	78 ± 9	39 ± 9	9 ± 2	0.92 ± 0.16	17.0	8.4

^a^ Values are means ± SD (n = 4); ^b^ Values are means ± SD (n = 12).

Recovery (%) is shown for different amounts of plant matrix together with determinations of the analytical precision and accuracy of whole procedure. Plant tissues (1–5 mg FW **of*A. thaliana*seedlings** spiked with 1 pmol of authentic CK standards) were extracted **in Bieleski buffer**, purified by **multi-StageTip microcolumn** chromatography and directly analyzed by UHPLC-ESI(+)-MS/MS.

**Table 4:** Recovery (%) of different CK groups in relation to number of sorbent multi-layers (C18, SDB-RPS, Cation-SR) used in PT-SPE purification procedure

	**Recovery (%)**^**a**^		
**CKs**	**Number of sorbent multi-layers a**		
	**1**	**2**	**3**
Free Bases	23 ± 3	58 ± 13	73 ± 15
Ribosides	11 ± 4	33 ± 9	77 ± 18
*N-*glucosides	7 ± 1	36 ± 23	85 ± 5
*O-*glucosides	7 ± 3	31 ± 21	81 ± 10
Nucleotides	5 ± 3	13 ± 2	81 ± 6

a Values are means ± SD (n = 4).

Plant tissues (5 mg FW of *A. thaliana* seedlings spiked with 1 pmol of authentic CK standards) were extracted in Bieleski buffer, purified by StageTip protocol and directly analyzed by UHPLC-ESI(+)-MS/MS.

**Table 5:** **Cytokinin levels in*Arabidopsis thaliana*extracts determined by UHPLC-ESI(+)MS/MS**

**CKs**	**Cytokinin content (pmol g**^**–1**^**FW)**			
	**Seedlings**	**Shoots**	**Roots**	**Root segments**
*t*Z	n.d.	n.d.	0.96 ± 0.12	2.60 ± 0.97
*t*ZR	2.23 ± 0.37	0.88 ± 0.32	3.27 ± 0.52	4.92 ± 0.94
*t*Z7G	37.99 ± 2.81	23.49 ± 3.07	6.54 ± 0.75	n.d.
*t*Z9G	3.88 ± 1.22	4.32 ± 1.31	2.08 ± 0.52	n.d.
*t*ZOG	9.25 ± 2.77	9.42 ± 1.75	7.10 ± 1.33	7.63 ± 2.45
*t*ZMP	n.d.	n.d.	n.d.	8.04 ± 2.44
*cZ*	n.d.	n.d.	n.d.	0.95 ± 0.33
*c*ZR	0.80 ± 0.16	1.14 ± 0.37	3.71 ± 0.79	2.28 ± 0.41
*c*Z9G	n.d.	n.d.	2.02 ± 0.47	n.d.
*c*ZOG	0.90 ± 0.27	1.04 ± 0.33	2.89 ± 0.66	3.35 ± 0.39
DHZR	0.64 ± 0.19	0.94 ± 0.27	0.97 ± 0.28	n.d.
DHZ7G	5.48 ± 1.28	5.78 ± 1.51	2.07 ± 0.42	n.d.
DHZ9G	n.d.	0.71 ± 0.22	0.29 ± 0.07	n.d.
DHZOG	0.46 ± 0.13	0.28 ± 0.07	0.15 ± 0.05	n.d.
iP	0.24 ± 0.10	0.15 ± 0.04	0.56 ± 0.17	0.42 ± 0.14
iPR	1.96 ± 0.28	1.24 ± 0.26	2.18 ± 0.46	5.15 ± 0.60
iP7G	53.87 ± 2.86	65.76 ± 12.47	30.16 ± 4.57	n.d.
iPMP	n.d.	n.d.	n.d.	13.14 ± 0.35

Values are meansf ± SD (n = 12); n.d. – not detected.

10-day-old *A. thaliana* seedlings, roots and shoots and apical part of the root of 8-day-old plants (approximately 0.5 cm) were extracted in Bieleski buffer, purified by multi-StageTip microcolumns and measured by UHPLC-ESI(+)-MS/MS method.

The precision and accuracy of the method, expressed as relative standard deviation (RSD) and as percentage bias, respectively, were also determined using acquired data from the non-spiked and spiked samples. Simultaneously, recoveries and intra- and interday relative standard deviations were calculated with CK standards spiked in Arabidopsis seedling extract at three different concentrations (0.1, 1 and 10 pmol mg^–1^). The cytokinin analytes were quantified by standard isotope dilution analysis [45], and subsequently the detected amounts of endogenous compounds were subtracted and the values were finally averaged for each compound. The mean precision was 11.2% (range 4.3–18.1% RSD), and the mean accuracy was 3.0%, ranging from −18.2 to 17.6% bias (Table 3). For low, medium and high CK concentrations, the relative recoveries were between 24% and 94%, and the intra- and interday variability did not exceed 10.2% and 12.9%, respectively ( Additional file 3). In general, the precision and accuracy tended to become worse when higher amounts of plant tissues were applied onto StageTips. Similar to our results published for UHPLC-ESI-MS/MS analyses of the different phytohormone groups [13,34], the acquired data showed that the variations in recoveries, validated by the internal standardization procedure, facilitated the detection of natural cytokinins within ±20% of the true amounts. Hence, accuracy of the developed analytical approach is satisfactory for determining these trace components in a complex plant matrix [14].

### Cytokinin profiling in minute amounts of Arabidopsis tissues

Finally, the newly developed and validated micropurification procedure, linked to highly selective and sensitive MS/MS detection, was applied for cytokinin isolation and analysis from the model plant *A. thaliana*. Addition of stable isotope-labelled standards as internal tracers during the extraction procedure allowed calculation of the intrinsic levels of isoprenoid cytokinins in minute amounts of *A. thaliana* seedlings, roots and shoots (1.0, 2.0, and 5.0 mg FW). Eighteen isoprenoid cytokinin metabolites were detected and quantified in a single unique UHPLC-ESI(+)-MS/MS run after microextraction and StageTip purification of the samples prepared in quadruplicates. In Table 5, the data are summarized as average values obtained for each tissue, recalculated per gram of fresh plant material. The detailed results for different sample loads of the extracted Arabidopsis roots and shoots as well as whole seedlings are presented in Additional file 4. Non-significant variations were seen in the estimated levels of the analytes which were on average 2.1(±0.5)-fold and 5.7(±1.6)-fold higher for 2 and 5 mg samples, respectively. The results corroborate the consequence of using specific labelled analogues as recovery markers for each hormone metabolite measured [46]. In summary, the measured amount of CKs for all tested plant tissues varied between 0.15 to 65.76 pmol g^–1^ FW. The wide spectrum of cytokinins obtained from the seedling samples was consistent with previously published profiles [13]. A similar pattern was found in the shoots and roots in agreement with Jones et al. [47]. The ratio between *cis-* and *trans*-zeatin-type CKs was similar to previously reported levels in the youngest leaves of *A. thaliana*[48]*.* In agreement with previously reported results [49,50], we have found the iP-type and *trans*-Z-type metabolites as the major cytokinins, comprising ca. 50% and 40% of the total CK content in *A. thaliana* tissues, respectively. Conversely, the different proportion of *cis-*Z-type CKs were found in the roots compared with CK profile in shoot samples analyzed (Table 5). Furthermore, the 7-glucoside forms, iP7G and *t*Z7G, were the most abundant CK metabolites in whole 10-day old seedlings as well as in shoot and root parts of the studied plants, followed by *O-*glucoside and 9-glucoside forms, which were present at approximately 10-fold lower concentrations. In accordance with our findings, Gajdošová et al. [48] noted the *N-* or *O-*glucosides as prevailing CK forms within more than 150 representative plant species analyzed. Moreover, the Arabidopsis roots contained higher (ca. 3-fold) levels of the bioactive CK forms (iP, *t*ZR, *c*ZR, DHZR, and iPR) than the shoots. No endogenous CK nucleotides were detected in whole 10-day-old *A. thaliana* seedlings samples, in comparison with the results obtained for the root segments (0.5 cm) of 8-day-old plants, where intact monophosphates of tZ and iP were detected and successfully quantified (see Table 5).

**Figure 5:**
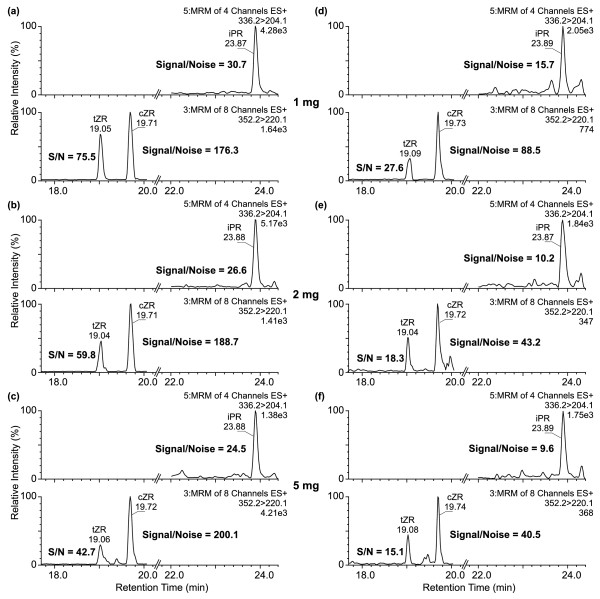
**Matrix effects on selectivity and sensitivity of UPLC–ESI(+)-MS/MS method.** MRM chromatograms of authentic t/cZR and iPR show the change in signal sensitivity for different A. thaliana extracts, roots (**a-c**) and shoots (**d-f**), in minute amounts of plant tissue (1, 2, and 5 mg FW).

In addition, the results also showed that the MS signal acquired for each CK metabolite was proportional to the amount and the type of purified plant material (Figure 5). Both selected tissues, the root and shoot systems, represented the samples with different complexity, proportions of potentially interfering compounds, and concentrations of lipids and plant pigments. In relation to the increasing sample weight, the purification and analysis of the extracts resulted in more stable signal-to-noise ratio for the root extracts (Figure 5a, b and c) in comparison with decreasing signal intensity in the shoot extracts (Figure 5d, e and f), respectively. This could be related to the higher presence of contaminants and impurities in some plant organs such as leaves, buds, stems, and flowers. Furthermore, the trends observed for recoveries of the internal standards added to the shoot samples (on average ca. 75% IS for 1.0 mg FW, 46% IS for 2.0 mg FW, and 16% IS for 5.0 mg FW recovered) agreed with our measured results for the spiked seedlings (Table 3). On the other hand, for Arabidopsis root extracts, the purification procedure yielded somewhat higher recoveries of ca. 98%, 58%, and 29% IS for 1.0 mg, 2.0 mg, and 5.0 mg FW, respectively. All data demonstrated that the effect of plant matrix grew rapidly with increasing amounts of plant tissues and showed the following progression among plant tissue types, seedlings > shoots > > roots. In conclusion, the advantages of using StageTip purification prior to MS analysis of CK derivatives, as well as other phytohormones, depend on the right choice of SPE sorbent used, in correlation with sample type, optimal amount of tissue extracted, and high sensitivity of the detection methods, respectively.

## Conclusions

To our knowledge, this is the first time that a STop And Go Extraction Tip purification procedure has been described for the determination of biologically active compounds in plant extracts. The data presented in this study demonstrated that the most common problems in analysis of phytohormones, namely occurrence of the plant hormones in minute quantities together with large amounts of interfering compounds, can be solved by the application of a miniaturized purification method linked to the high separation efficiency and sensitivity of UHPLC-MS/MS analysis. The combination of microextraction with one-step high-throughput solid-phase extraction in StageTips provides fast, effective and cheap sample purification prior to qualitative and quantitative measurements. The pipette tip SPE can be tested (after some modifications) also for other phytohormones, depending on selectivity, affinity and capacity of the selected sorbents. Use of our procedure can allow the quantification of plant hormones in very limited amounts of material, and can be beneficial for samples such as root tips, meristems and embryos.

## Methods

### Reagents and materials

Authentic and deuterium-labelled CK standards were obtained from Olchemim Ltd (Olomouc, Czech Republic). Tritium-labelled CK standards ([^3^H]*t*ZR, 0.851 TBq mmol^−1^; [^3^H]*c*Z, 0.777 TBq mmol^−1^; [^3^H]iPR, 1.184 TBq mmol^−1^) with radiochemical purity >98% were synthesized in the Isotope Laboratory, Institute of Experimental Botany, AS CR (Prague, Czech Republic). Methanol (gradient grade), formic acid and ammonium hydroxide were from Merck (Darmstadt, Germany); chloroform, acetone, nitric acid, acetic acid and formic acid for LC/MS from Sigma-Aldrich (St. Louis, MO, USA); Murashige & Skoog medium from Duchefa Biochemie B.V. (Haarlem, The Netherlands); Empore^TM^ High Performance Extraction Disks (sorbent type C18, SDB-RPS and Cation-SR) from 3M Center (St. Paul, MN, USA). Deionized (Milli-Q) water was obtained from Simplicity 185 system (Millipore, Bedford, MA, USA) and scintillation cocktail Ultima Gold^TM^ was from Packard BioScience (Groningen, The Netherlands).

### Plant material and growth conditions

*Arabidopsis thaliana* seedlings (ecotype Colombia) were grown *in vitro* in Petri dishes containing Murashige & Skoog medium including vitamins (4.4 g MS medium, 10 g of sucrose, 10 g of plant agar l^–1^, pH 5.7) at 23°C in a 16 h photoperiod. 10-day-old seedlings, roots and shoots were harvested, weighed and immediately plunged into liquid nitrogen. Moreover the apical part of the root of 8-day-old seedlings (approximately 0.5 cm of root length) was collected, weighed, and frozen in liquid nitrogen. All samples were stored at −70°C.

### Pipette tip SPE purification

Bieleski buffer (60% methanol, 25% CHCl_3_, 10% HCOOH and 5% H_2_O) was used as extraction solvent (50 μl per sample). Tritium-labelled (10^5^ dpm) or unlabelled (1 pmol) cytokinin standards were added to the sample extracts during the method optimization to determine the recoveries of the StageTip purification procedure. To validate the quantification of the endogenous cytokinin levels in *A. thaliana* seedlings, roots and shoots, the following stable isotope-labelled cytokinin internal standards (IS) were added as internal tracers at a concentration 0.5 pmol of each compound per 50 μl of Bieleski buffer: [^13^C_5_]*c*Z, [^13^C_5_]*t*Z, [^2^H_5_]*t*ZR, [^2^H_5_]*t*Z7G, [^2^H_5_]*t*Z9G, [^2^H_5_]*t*ZOG, [^2^H_5_]*t*ZROG, [^2^H_5_]*t*ZMP, [^2^H_3_]DHZ, [^2^H_3_]DHZR, [^2^H_3_]DHZ9G, [^2^H_7_]DHZOG, [^2^H_3_]DHZMP, [^2^H_6_]iP, [^2^H_6_]iPR, [^2^H_6_]iP7G, [^2^H_6_]iP9G, [^2^H_6_]iPMP. The plant material was placed in 2.0 ml microcentrifuge tubes and extracted in Bieleski solvent using a MM 301 vibration mill (Retsch GmbH & Co. KG, Haan, Germany) at a frequency of 27 Hz for 3 min after adding 3 mm tungsten carbide beads (Retsch GmbH & Co. KG, Haan, Germany) to increase the extraction efficiency. The tube content was ultrasonicated for 3 min and then stirred for 30 min at 4°C. After centrifugation (10 min, 15,000 rpm, 4°C) the supernatants (50 μl aliquots) were immediately transferred onto StageTips and purified according to the following protocol.

The PT-SPE was performed in self-packed StageTips by placing a very small disk of matrix in an ordinary pipette tip. Commercially available matrix of poly-tetrafluoroethylene containing reversed-phase octadecyl-bonded silica phase (C18) or poly(styrene-divinylbenzene) (SDB) copolymer modified with sulfonic acid groups to make it more hydrophilic (SDB-RPS Disk) was normally used. Alternatively, ion-exchange sorbent including sulfonic acid as cation exchanger (Cation-SR Disk) was also employed. The procedure shown in Additional file 5 was described by Rappsilber et al. [23,26]. Small disks (approximately 1.0 mm diameter, 0.5 mm thickness) were cut out manually from the Empore^TM^ High Performance Extraction Disk placed on a clean surface (Petri dish) using a hollow tool cutter (blunt-ended syringe needle). The cutter was gently pressed into the Empore disk and the material penetrated to the inside of the needle. Subsequently, the cutter was placed inside a pipette tip (disposable GELoader® Tip, 100 μl, from Eppendorf). The small disk was then released using a plunger (rod) that fitted into the needle (both parts from Hamilton) and pressed gently repeatedly into place using the weight of the plunger. After removing the cutter and plunger, the single-StageTip was finished. Additional disks were added the same way to produce combined multi-StageTips.

The Empore sorbents were tested individually (C18, SDB-RPS, and Cation-SR) or in combination (C18/SDB-RPS, C18/Cation-SR, C18/SDB-RPS/Cation-SR). All solutions were loaded from the top of the tip in a volume of 50–100 μl using a pipette. The prepared StageTip was inserted into a hole at the centre of the lid of the microcentrifuge tube (1.5 ml) and placed in a centrifuge after solvent pipetting (Figure 2). Prior to loading the sample the StageTip sorbents were activated with 50 μl acetone (by centrifugation at 1,500 rpm, 15 min, 4°C), 50 μl water (1,500 rpm, 15 min, 4°C), 50 μl methanol (1,500 rpm, 15 min, 4°C), 50 μl water (1,500 rpm, 15 min, 4°C), equilibrated with 50 μl 50% (v/v) nitric acid (1,000 rpm, 20 min, 4°C), 50 μl water (1,500 rpm, 15 min, 4°C) and 50 μl Bieleski solvent (1,500 rpm, 15 min, 4°C). Afterwards, the samples were loaded in extraction buffer (500 rpm, 45 min, 4°C). The tips were washed using 50 μl methanol (1,500 rpm, 15 min, 4°C) and elution of samples was performed with 50 μl of 0.5 M NH_4_OH in 60% MeOH (500 rpm, 45 min, 4°C). Eluates were collected into new clean microcentrifuge tubes and directly mixed with scintillation buffer prior to measurement of radioactivity or evaporated to dryness in a Speed-Vac concentrator RC1010 (Jouan, Winchester, UK) and dissolved in 20 μl of mobile phase prior to UHPLC-MS/MS analyses.

### Instrumentation

The radioactivity of tritium-labelled cytokinin standards was measured after addition of 1 ml liquid scintillation cocktail Ultima Gold^TM^ on a multi-purpose scintillation counter LS 6500 (Beckman Coulter, Brea, CA, USA). An Acquity UPLC® System (Waters, Milford, MA, USA), including a Binary solvent manager and Sample manager was linked simultaneously to a 2996 PDA detector (Waters) and a triple quadrupole mass spectrometer Xevo^TM^ TQ MS (Waters MS Technologies, Manchester, UK) equipped with an electrospray interface (ESI) and the unique performance of a collision cell (ScanWave^TM^). This hyphenated technique was used for analysis of unlabelled and stable isotope-labelled cytokinins. All MS data were processed by MassLynx^TM^ software with TargetLynx^TM^ program (version 4.2., Waters, Milford, MA, USA).

### UHPLC-ESI(+)-MS/MS conditions

The samples pre-purified on StageTips were dissolved in 20 μl of 10% methanol and 10 μl of each sample was injected onto a reversed-phase column (Acquity UPLC® BEH C18, 1.7 μm, 2.1 × 150 mm, Waters). The samples were eluted with a 24-min gradient composed of methanol (A) and 15 mM ammonium formate pH 4.0 (B) at a flow rate of 0.25 ml min^−1^, column temperature of 40°C and sample temperature of 4°C. The following binary gradient was used: 0 min, 5:95 (A:B) – 7.0 min isocratic elution, 5:95 (A:B) – 9.0 min linear gradient, 20:80 (A:B) – 7.0 min linear gradient, 50:50 (A:B) – 1 min isocratic elution, 50:50 (A:B). At the end of the gradient the column was washed with 100% methanol and re-equilibrated to initial conditions (6 min). The effluent was passed through an ultraviolet-diode array detector (scanning range 200–400 nm, resolution 1.2 nm, sampling rate 5 points s^−1^) and the tandem mass spectrometer Xevo TQ MS without post-column splitting. Selective multiple reaction monitoring (MRM) mode using mass-to-charge (m/z) transitions of precursor and product ions was performed under MS conditions optimized earlier [13] with few modifications: capillary voltage 0.35 kV; source/desolvation gas temperature 120/575°C; cone/desolvation gas flow rates 70/1000 l hr^−1^; collision gas (argon) pressure/flow 5 10^−3^ mbar/0.2 ml min^−1^; LM/HM resolution 2.8/15.0; ion energy 1/2 1.0 V; entrance/exit voltages 0.5 V, respectively. The MS/MS parameters like dwell time (automatic mode for 16 scan points per peak), cone voltage (21–35 V) and collision energy (15–24 eV) were selected to maximize the sensitivity of exact diagnostic transitions. Quantification was conducted using a standard isotope dilution method. The ratio of endogenous cytokinin to the appropriate labelled standard was determined and further used to quantify the level of endogenous compounds in the original extract, according to a known quantity of an added internal standard [10]. The calibration curves ranged 100 amol to 100 pmol were constructed by serial dilutions of the CK standards listed in Figure 1 and the known concentration of the appropriate internal labelled standards.

### Validation of the analytical procedure

To test the selectivity, affinity, and capacity of different StageTip sorbent combinations, samples containing ^3^H-labelled CK standards ([^3^H]*c*Z, [^3^H]*t*ZR, [^3^H]iPR) were used. Firstly, to characterize the potential sorbents (C18, SDB-RPS, and Cation-SR), the single-StageTip experiments were performed by pipetting 50 μl of the selected ^3^H-CK standard (10^5^ dpm) in Bieleski buffer directly onto a PT-SPE column. Secondly, different combinations of sorbents (multi-StageTips) were tested using the same spiked Bieleski buffer with and without plant matrices (1.0 and 2.0 mg FW) to monitor the loading capacity and extraction recovery. For all samples, the yields of ^3^H-CKs after passage through StageTips filled with different sorbents and their combinations were determined. Thirdly, non-spiked and spiked (with 0.1, 1 and 10 pmol of unlabelled CK standards) samples of 10-day old *Arabidopsis thaliana* seedlings (1–5 mg FW) were used to verify the reproducibility, sensitivity and accuracy of the method. Aliquots of the extracts were processed using the developed purification procedure (Figure 2), then analyzed by UHPLC-ESI(+)-MS/MS. The intraday reproducibility was evaluated by repeating the process for three times within one day, and the interday reproducibility was investigated on three successive days. The recovery of added authentic cytokinin standards were then determined from each series of extracts, based on the amounts of endogenous compounds, calculated from non-spiked samples, which subsequently served as reference levels.

## Abbreviations

C18, Octadecyl-bonded silica phase; CK, Cytokinin; ESI, Electrospray interface; IS, Internal standards; LOD, Limit of detection; LOQ, Limit of quantification; MRM, Multiple reaction monitoring; MS/MS, Tandem mass spectrometry; PT-SPE, Pipette tip solid-phase extraction; RPS, Reversed-phase sorbent; RSD, Relative standard deviation; SDB, Poly(styrene-divinylbenzene); StageTip, STop And Go Extraction; UHPLC, Ultra-high performance liquid chromatography.

## Competing interests

The authors declare that they have no competing interests.

## Authors’ contributions

JS, ON, RL and KD designed the research, JS, ON, LP, and JH performed the research, JS, ON, LP and RL analyzed the data and JS, ON, MS and KD wrote the paper. All authors read and approved the final manuscript.

## Supplementary Material

Additional file 1 **The loading capacity and extraction recovery of C18/SDB-RPS/Cation-SR microcolumns.** Different steps of purification protocol are described in **Figure** 2**.** Tritium-labelled standards (10^5^ dpm) were dissolved in Bieleski buffer and the extracts without (a; 0 mg FW) and with plant matrix (b; 2 mg FW) were applied onto multi-StageTips. Values are means ± SD (n = 4); [^3^H]*c*Z, black bars; [^3^H]*t*ZR, white bars; [^3^H]iPR, square bars. Click here for file

Additional file 2 **Identification of (−)-DHZ7G with (S)-configuration in *A. thaliana *extracts.** Identification based on accordance with the peak retention time of reference standard mixture (a) and different plant extracts (b-c) injected onto an Acquity UPLC® BEH C18 2.1 × 150 mm column using the selective MRM transition (384.2 > 222.1). (a) Representative MRM chromatograms of racemic (±)-DHZ7G standard; (b) Arabidopsis shoot extract; (c) Arabidopsis root extract. The samples (2 mg FW) were extracted in Bieleski buffer, purified by StageTip protocol (**Figure** 2) and measured by UHPLC-ESI(+)-MS/MS method under experimental conditions (Chapter 2.5). Click here for file

Additional file 3 **Method validation.** Intra- and interday precisions (%RSD) and recovery (%) is shown for three different concentrations (Low – 0.1 pmol mg^–1^; Medium – 1 pmol mg^–1^; High – 10 pmol mg^–1^). Plant tissues (1 mg FW of *A. thaliana* seedlings spiked with mixture of authentic CK standards) were extracted in Bieleski buffer, purified by multi-StageTip microcolumn chromatography and directly analyzed by UHPLC-ESI(+)-MS/MS. Click here for file

Additional file 4 **Levels of CK metabolites in varied amounts of *Arabidopsis thaliana *seedlings, roots and shoots. ** 10-day-old seedlings, roots and shoots were extracted in Bieleski buffer, purified by StageTip microcolumns and measured by UHPLC-ESI(+)-MS/MS method. *t*/*c*Z, *t*/*c*ZROG, *t*/*c*ZMP, DHZ, DHZROG, DHZMP, iP9G, and iPMP were not detected in Arabidopsis extracts. Click here for file

Additional file 5 **Step-by-step guide on the preparation of a StageTip.** (1) The pipette tip, Empore^TM^ High Performance Extraction Disk placed on a Petri dish, cutter (blunt-ended syringe needle) and plunger (rod needle); (2) cutting of the small disk (approximately 1.0 mm diameter, 0.5 mm thickness); (3–7) insertion of the disk into the pipette tip using the cutter and plunger fitted into the needle; (8) placing of additional disk onto the first disk; (9) single-StageTip (on the left) and multi-StageTips (on the right). Click here for file
