# Supplementary figures and images for: A new approach for cytokinin isolation from Arabidopsis tissues using miniaturized purification: pipette tip solid-phase extraction
Source: Plant Methods. 2012 May 17;8:17. doi: 10.1186/1746-4811-8-17 (PMC3492005; doi:10.1186/1746-4811-8-17)

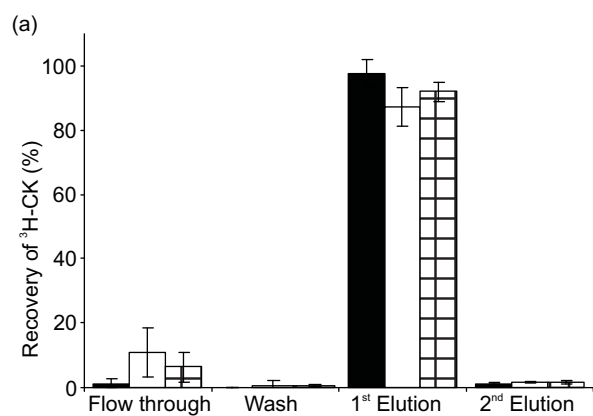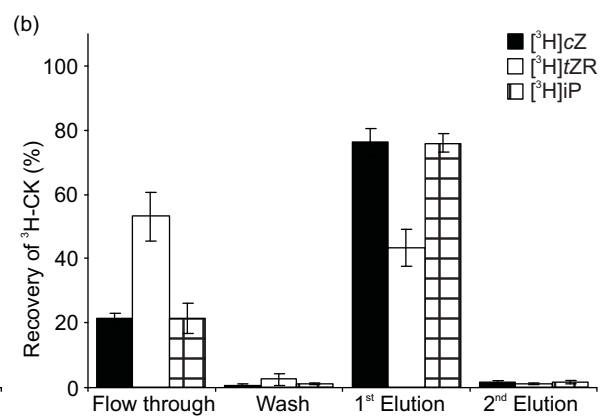

Supplement: Additional file 2 — Identification of (−)-DHZ7G with (S)-configuration in A. thaliana extracts. Identification based on accordance with the peak retention time of reference standard mixture (a) and different plant extracts (b-c) injected onto an Acquity UPLC® BEH C18 2.1 × 150 mm column using the selective MRM transition (384.2 > 222.1). (a) Representative MRM chromatograms of racemic (±)-DHZ7G standard; (b) Arabidopsis shoot extract; (c) Arabidopsis root extract. The samples (2 mg FW) were extracted in Bieleski buffer, purified by StageTip protocol (Figure 2) and measured by UHPLC-ESI(+)-MS/MS method under experimental conditions (Chapter 2.5). [file 1746-4811-8-17-S2.pdf]

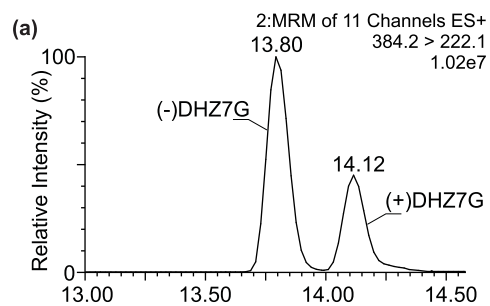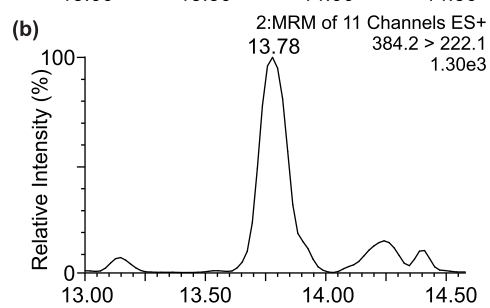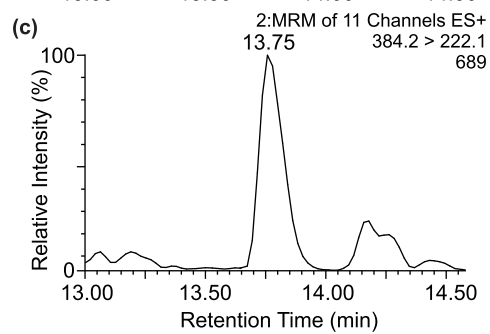

Supplement: Additional file 3 — Method validation. Intra- and interday precisions (%RSD) and recovery (%) is shown for three different concentrations (Low – 0.1 pmol mg–1; Medium – 1 pmol mg–1; High – 10 pmol mg–1). Plant tissues (1 mg FW of A. thaliana seedlings spiked with mixture of authentic CK standards) were extracted in Bieleski buffer, purified by multi-StageTip microcolumn chromatography and directly analyzed by UHPLC-ESI(+)-MS/MS. [file 1746-4811-8-17-S3.pdf]
