# Supplementary material for: A new approach for cytokinin isolation from Arabidopsis tissues using miniaturized purification: pipette tip solid-phase extraction
Source: Plant Methods. 2012 May 17;8:17. doi: 10.1186/1746-4811-8-17 (PMC3492005; doi:10.1186/1746-4811-8-17)
Supplement: Additional file 4 — Levels of CK metabolites in varied amounts of Arabidopsis thaliana seedlings, roots and shoots. 10-day-old seedlings, roots and shoots were extracted in Bieleski buffer, purified by StageTip microcolumns and measured by UHPLC-ESI(+)-MS/MS method. t/cZ, t/cZROG, t/cZMP, DHZ, DHZROG, DHZMP, iP9G, and iPMP were not detected in Arabidopsis extracts. [file 1746-4811-8-17-S4.pdf]

Additional file 4 - Method validation.

Intra- and interday precisions (%RSD) and recovery (%) is shown for three different concentrations (Low – 0.1 pmol/mg; Medium – 1pmol/mg; High – 10 pmol/mg). Plant tissues (1 mg FW of *A. thaliana* seedlings spiked with mixture of authentic CK standards) were extracted in Bieleski buffer, purified by multi-StageTip microcolumn chromatography and directly analysed by UPLC-ESI(+)-MS/MS.

CKs	Intraday precision[a]			Interday precision[a]			Recovery (%)[b]		
	L[c]	M[c]	H[c]	L	M	H	L	M	H
*t*Z	6.3%	8.4%	3.7%	12.0%	7.7%	10.4%	89 ± 7	80 ± 10	84 ± 5
*t*ZR	3.8%	5.4%	2.6%	8.1%	9.0%	6.6%	73 ± 1	72 ± 12	82 ± 5
*t*Z7G	3.4%	2.2%	0.3%	5.1%	1.3%	0.3%	93 ± 2	88 ± 6	89 ± 7
*t*Z9G	9.0%	5.8%	3.4%	11.4%	8.6%	2.6%	78 ± 3	59 ± 7	84 ± 7
*t*ZOG	2.5%	1.6%	1.9%	8.0%	6.6%	6.5%	88 ± 10	85 ± 5	91 ± 5
*t*ZROG	7.4%	8.2%	8.7%	5.7%	8.2%	5.1%	58 ± 11	55 ± 4	61 ± 2
*t*ZMP	10.2%	2.0%	4.5%	11.9%	3.1%	5.7%	40 ± 12	35 ± 6	29 ± 4
*c*Z	9.2%	6.0%	2.9%	11.7%	7.1%	10.3%	86 ± 9	75 ± 9	86 ± 11
*c*ZR	8.7%	2.3%	2.1%	5.2%	8.2%	6.3%	83 ± 4	81 ± 13	94 ± 7
*c*Z9G	9.2%	2.0%	2.4%	6.1%	6.6%	0.7%	78 ± 6	74 ± 12	82 ± 7
*c*ZOG	8.2%	7.4%	2.8%	9.1%	5.3%	7.2%	92 ± 3	89 ± 6	96 ± 2
*c*ZROG	6.2%	5.4%	4.1%	8.5%	12.5%	12.1%	65 ± 4	52 ± 6	58 ± 5
*c*ZMP	9.2%	7.7%	6.7%	11.7%	8.1%	9.3%	24 ± 5	32 ± 3	30 ± 3
DHZ	8.3%	4.4%	0.7%	6.1%	4.1%	5.1%	74 ± 6	77 ± 13	81 ± 5
DHZR	0.7%	1.1%	2.8%	7.0%	4.2%	2.0%	74 ± 6	88 ± 13	92 ± 13
DHZ7G	4.0%	2.8%	1.1%	4.5%	1.0%	1.4%	94 ± 2	89 ± 3	86 ± 9
DHZ9G	6.2%	3.6%	2.3%	4.3%	5.5%	5.3%	90 ± 9	78 ± 10	67 ± 10
DHZOG	5.9%	2.4%	1.8%	5.8%	6.5%	2.8%	91 ± 1	77 ± 5	87 ± 2
DHZROG	9.1%	4.5%	2.0%	12.9%	9.3%	5.0%	90 ± 2	87 ± 8	95 ± 5
DHZMP	10.1%	8.9%	9.1%	12.9%	10.7%	7.7%	45 ± 8	37 ± 1	40 ± 3
iP	2.6%	6.2%	2.4%	1.8%	5.5%	5.0%	66 ± 3	76 ± 9	86 ± 10
iPR	4.6%	2.6%	1.0%	3.5%	1.9%	1.5%	88 ± 2	84 ± 8	98 ± 8
iP7G	5.1%	3.1%	1.1%	3.4%	1.7%	0.9%	94 ± 2	83 ± 10	90 ± 8
iP9G	3.3%	2.7%	1.6%	11.9%	8.4%	5.0%	67 ± 11	74 ± 8	92 ± 8
iPMP	7.5%	4.1%	4.5%	9.8%	11.0%	7.6%	76 ± 17	78 ± 9	69 ± 9

[a] %RSD (n=3); [b] Values are means ± SD (n = 4); [c] L=0.1 pmol/mg, M=1pmol/mg; H=10 pmol/mg.
