# Supplementary material for: A new approach for cytokinin isolation from Arabidopsis tissues using miniaturized purification: pipette tip solid-phase extraction
Source: Plant Methods. 2012 May 17;8:17. doi: 10.1186/1746-4811-8-17 (PMC3492005; doi:10.1186/1746-4811-8-17)
Supplement: Additional file 5 — Step-by-step guide on the preparation of a StageTip. (1) The pipette tip, EmporeTM High Performance Extraction Disk placed on a Petri dish, cutter (blunt-ended syringe needle) and plunger (rod needle); (2) cutting of the small disk (approximately 1.0 mm diameter, 0.5 mm thickness); (3–7) insertion of the disk into the pipette tip using the cutter and plunger fitted into the needle; (8) placing of additional disk onto the first disk; (9) single-StageTip (on the left) and multi-StageTips (on the right). [file 1746-4811-8-17-S5.pdf]

Additional file 5 - Levels of CK metabolites in varied amounts of *Arabidopsis thaliana* seedlings, roots and shoots.

10-day-old seedlings, roots and shoots were extracted in Bieleski buffer, purified by StageTip microcolumns and measured by UPLC-ESI(+)-MS/MS method. *t*/*c*Z, *t*/*c*ZROG, *t*/*c*ZMP, DHZ, DHZROG, DHZMP, iP9G, and iPMP were not detected in Arabidopsis extracts.

Weight (mg FW)	Cytokinin content (fmol in sample)														
	***t*Z**	***t*ZR**	***t*Z7G**	***t*Z9G**	***t*ZOG**	***c*ZR**	***c*Z9G**	***c*ZOG**	**DHZR**	**DHZ7G**	**DHZ9G**	**DHZOG**	**iP**	**iPR**	**iP7G**
**Seedlings**															
1	n.d.	n.d.	41.9±1.9	3.7±1.1	8.3±2.5	0.6±0.1	n.d.	1.0±0.3	n.d.	5.6±1.1	n.d.	0.5±0.1	n.d.	1.7±0.2	51.1±2.0
2	n.d.	3.4±0.4	70.2±9.0	8.2±2.6	18.7±5.0	1.5±0.9	n.d.	1.5±0.3	0.9±0.5	10.4±3.4	n.d.	0.8±0.2	n.d.	3.7±0.6	105.0±4.2
5	n.d.	13.9±2.9	184.8±10.0	n.d.	50.9±6.6	3.2±0.3	n.d.	5.1±2.0	3.5±1.2	28.3±5.4	n.d.	2.7±0.9	1.2±0.6	11.6±2.0	270.1±22.7
**Shoots**															
1	n.d.	0.8±0.3	26.8±2.8	4.5±1.5	8.1±2.1	1.1±0.4	n.d.	1.1±0.4	0.8±0.2	6.2±2.0	0.7±0.3	0.3±0.1	n.d.	1.1±0.2	58.0±11.5
2	n.d.	1.6±0.6	44.2±3.8	10.7±3.7	17.9±2.4	2.0±0.8	n.d.	1.7±0.5	1.9±0.7	10.4±1.9	1.7±0.6	0.6±0.2	n.d.	2.3±0.4	127.6±21.9
5	n.d.	5.5±1.7	107.9±22.8	15.5±2.8	55.9±9.9	4.5±1.5	n.d.	5.8±1.9	5.5±1.6	29.6±7.7	2.9±0.6	1.2±0.3	0.7±0.1	7.2±2.0	312.4±74.7
**Roots**															
1	n.d.	3.7±0.6	7.3±0.6	1.9±0.6	6.3±0.5	3.1±0.8	1.3±0.2	3.1±0.9	0.6±0.2	2.2±0.3	0.3±0.1	n.d.	0.4±0.2	2.0±0.3	26.2±4.8
2	2.1±0.7	6.1±1.3	11.4±0.7	4.1±1.4	13.6±2.0	6.8±1.3	4.9±0.7	5.9±1.1	2.0±0.7	3.7±0.6	0.6±0.1	0.4±0.1	0.7±0.1	4.3±0.9	61.9±7.5
5	4.2±1.1	15.4±1.8	32.9±6.8	11.5±1.4	37.8±8.2	23.4±4.9	14.3±4.3	13.2±2.4	6.3±2.1	10.9±3.2	1.3±0.2	n.d.	2.0±0.7	12.1±3.1	166.9±26.1

Values are means ± SD (n = 4); n.d. – not detected.
